# Everybody's Page

**Published:** 1890-05-10

**Authors:** 


### Everybody's Page.

It is said that it takes one thousand rose trees to supply two ounces of attar of roses, and this amount is worth £20.

---

Strong newly-made coffee taken without milk or sugar has been found by some to be an excellent substitute for spirits. It ought to be a help to those who work for temperance, and who find the craving for spirits that a drunkard has so difficult to fight.

---

In the good old days it was the custom in some parts of the country to be bled on May Day, and it was a common idea that if a man were bled one year he must be bled the next and every succeeding year, for if it were left off he would be in great danger. The reason why we cannot tell; it was probably some clever old phlebotomist with a keen eye to business who originated the superstition.

---

A short time ago we mentioned the fact that a house had been made entirely of compressed paper; now the latest invention is flower vases of the same material. They are light, hard as iron, and retain none of the disagreeable stale smell left by flowers. Whether their light weight would cause them to be easily capsized is another question. They ought, however, if really successful to be a new idea for the Lady Decorative Artists, who could colour and paint them and make them look very pretty.

---

The Roman Catholic Church and the magistrates in the criminal courts are the two great opponents of the progress of cremation in France. The first cannot fight it on religious ground, for scripture nowhere forbids cremation, but, anyhow, the Church denies cremation to its members. The second opponent has, however, more argument to bring. The detection of crime by exhuming bodies and examining them would be put an end to. All we can hope is that a solution of this latter difficulty will be forthcoming very soon.

---

The largest fee Sir Astley Cooper ever received was literally thrown at his head. He operated very successfully on a millionaire, by name Hyatt, and so delighted was the old man with his recovery that he gave 300 pounds to each of his attending physicians. "But you, sir," cried the patient to Sir Astley, "deserve something better. Take that, sir!" With that he flung his nightcap at the surgeon. Sir Astley replied with dignity, as he picked up the cap, "Sir, I will pocket the affront," and well for him that he did, for the cap was lined with a draft for 1,000 guineas.

---

Last year we made an appeal in this paper to the ladies who attend the Queen's Drawing Rooms. We asked them to give something to add a little extra joy to those whose lives are saddened by sickness and suffering—the inmates of our hospitals. Our appeal was a very simple one; it was for flowers, the giving of which would be a small sacrifice in some cases, in others none at all. Could not the ladies spare their lovely bouquets and leave them behind in some room when they retire from Buckingham Palace, and her Majesty, always ready to do a kindness, would surely have them taken by her servants to different suitable institutions. Some fair débutantes will urge a ball in the evening to which they must carry their bouquet; others celebrate their first Drawing Room by being photographed in their finery, bouquet and all. But this is not the case with everybody. The delight a bouquet straight from the Queen's palace would cause in a ward is no enthusiastic illusion.

---

Since the opening of the Pasteur Institute in New York on February 18th up to March 31st, thirty-one persons had applied for relief.

---

Both patients and consulting physicians should lay the following to heart:—

> "A single Doctor like a sculler plies,
> The patient lingers and by inches dies;
> But two physicians like a pair of oars,
> Waft him with swiftness to the Stygian shores."

---

The population of Spain has increased in the last thirteen years at the rate of 0·54 per cent. This increase is considered to be due to the public tranquillity and the better hygienic condition of the towns. It is noticed that in those parts of the country where means of communication are practically nil the population has remained stationary, but it has risen proportionately in those parts where development and industry of commerce are thriving.

---

The food of the Turkish labourer, though frugal, is at least wholesome. In the towns his food in the summer consists during the day of a loaf of bread, with the addition of a little fruit. Before he goes to rest he has a dish of vegetables. In the winter an onion, a few olives, or a slice of cheese take the place of the fruit. His drink is black coffee, of which he does not stint himself. Meat is seldom taken, excepting on grand feast days. Here is a large community almost entirely given up to vegetarianism.

---

The exhibition of home-made silks which was opened at Lord Egerton's house in St. James's Square during the present week is to demonstrate the superiority of English silks over those of foreign manufacture. Irish silks and poplins are amongst the exhibits. We hear many people wishing that home trade would improve, and such an exhibition ought to do good; but would not much more benefit arise if purchasers of new materials would ask for and buy those of home manufacture? For in these days of the "Merchandise Marks Act" this would entail no extra trouble.

---

In all the rush and hurry that necessarily accompany the London season there are so many tickets for entertainments thrown away unused. Ladies want to help the giver of a concert and so they buy tickets for it, but other engagements prevent them attending, and away go the tickets into the waste paper basket. Zoological and Botanical Garden passes are also very nice, but are often made no use of. Nurses in the hospitals would be very glad if some of these stray tickets found their way to the matron, who could give them out at her discretion.

---

Now is the time of proficiency in everything; the days of second-rate performance are over, or are supposed to be, and technical schools flourish, and certificates of merit are the order of the day. Why, now that education marches, has nobody ever tried to improve street music? Why should not "German bands" have a certificate of merit, and only be allowed to sound in the far too tolerant British public ears when they possess some small degree of excellence? This old worn subject means nothing to thousands, but it means oceans to the tired brain-worker who knows that when he hears the first insinuating note of a cornet come round the corner he must put by his work, for in a second the whole brass band bursting into a blatant roar (each musician (?) playing in a different key) will blow every idea and thought into atoms. Oh! that some philanthropist would turn his attention to brass bands.

